# Neutralizing antibody durability and SARS-CoV-2 infection in older adults six months after XBB-containing vaccine booster

**DOI:** 10.1038/s41392-025-02437-y

**Published:** 2025-10-09

**Authors:** Rui-Rui Chen, Guo-Ping Cao, Xue-Dong Song, Mei Lu, Ming-Ming Wang, Xue-Jun Wang, Meng-Fei Wang, Shuang-Qing Wang, Sheng Wan, Guo-Jian Yang, Lei Lv, Yi-Ming Ma, Yi-Man Cheng, Meng Kong, Xue-Juan He, Hai-Yan Yang, Bing-Dong Zhan, Mai-Juan Ma

**Affiliations:** 1 School of Public Health, Zhengzhou University, Zhengzhou, China; 2 State Key Laboratory of Pathogen and Biosecurity, Academy of Military Medical Sciences, Beijing, China; 3 Department of Infectious Disease Control and Prevention, Quzhou Center for Disease Control and Prevention, Quzhou, China; 4 Department of Laboratory Medicine, Handan Central Hospital, Hebei Medical University, Handan, China; 5 Kaihua Center for Disease Control and Prevention, Quzhou, China; 6 Bioinformatics Center of the Academy of Military Medical Science, Beijing, China; 7 School of Public Health, Hebei Medical University, Shijiazhuang, China; 8 Department of Microbiological Laboratory Technology, School of Public Health, Cheeloo College of Medicine, Shandong University, Jinan, China; 9 School of Public Health, Nanjing Medical University, Nanjing, China

**Keywords:** Translational research, Vaccines, Infectious diseases

## Abstract

The persistence of XBB-containing vaccine-induced immunity against evolving SARS-CoV-2 variants remains uncertain, particularly in older adults, who are at increased risk of severe outcomes and may experience more rapid immune decline. We previously reported neutralizing antibody (nAb) responses 21 days after a single booster dose of trivalent XBB.1.5 (Tri‑XBB.1.5), bivalent Omicron XBB (Bi‑Omi‑XBB), or tetravalent XBB.1 (Tetra‑XBB.1) vaccines in a cohort of 90 older adults aged >65 years. In this six-month longitudinal follow-up analysis of the same cohort, we assessed nAb durability and SARS-CoV-2 infections to extend our earlier findings. Six months post-vaccination, the nAb titers decreased over time, with 2.5‒4.6-fold reductions in the geometric mean titer against the variants KP.3.1.1 and XEC; however, the nAb titer remained detectable in most participants. The Tri‑XBB.1.5 vaccine exhibited marginally better antibody persistence than the Bi‑Omi‑XBB and Tetra‑XBB.1 vaccines did, suggesting potential formulation-dependent differences in long-term immunogenicity. Twenty-seven participants experienced SARS-CoV-2 infection, including 11 (12.2%) confirmed by rapid antigen testing and 16 (17.8%) classified as probably based on serological evidence, with relatively frequent infections in bivalent and tetravalent recipients. Lower nAb titers on day 21 were linked to early infections, whereas late infections reflected antibody waning. Infections transiently increased nAb levels, but the elevated titers were not sustained. These findings highlight the importance of updating booster formulations and improving vaccine designs to address emerging immune-evasive variants.

## Introduction

Since the World Health Organization (WHO) declared the coronavirus disease 2019 (COVID-19) pandemic caused by severe acute respiratory syndrome coronavirus 2 (SARS-CoV-2) in March 2020, nearly five years have passed.^1^ Although the WHO announced the end of the public health emergency phase of the COVID-19 pandemic in May 2023, COVID-19 continues to pose a significant global health challenge.^2^ As of March 11, 2025, the pandemic has claimed over 7 million lives worldwide.^3^ Since the beginning of the pandemic, the development of vaccines has been a high priority in controlling the pandemic. Multiple vaccine platforms, including inactivated, mRNA, vector, and protein-based platforms,^4^ have been developed to target the ancestral Wuhan-Hu-1 strain of SARS-CoV-2, which represents a significant milestone in the global response to the pandemic by substantially reducing disease severity, hospitalizations, and mortality, even though protection against infection itself has varied between variants and over time.^5,6^

The dynamic evolution of SARS-CoV-2 has been a central challenge for sustaining protection. Beginning in April 2020, a series of SARS-CoV-2 variants of concern emerged within the span of a year, including D614G (B.1.1.7), Alpha (B.1.1.7), Beta (B.1.351), Gamma (P.1), and Delta (B.1.617.2).^7^ These variants are characterized by mutations that increase viral fitness, transmissibility, and immune evasion.^6–8^ Owing to the increased resistance of these variants to vaccine-induced neutralizing antibodies (nAbs), the effectiveness of vaccines targeting the ancestral Wuhan-Hu-1 strain has significantly decreased.^6–10^ In response, variant-specific vaccines have been developed to restore protection against these evolving strains.^11–19^ However, the rapid evolution of SARS-CoV-2 continued to challenge these updated vaccines, with the emergence of the highly transmissible Omicron variant BA.1 (B.1.1.529) in mid-November 2021, which represented a major antigenic shift and exhibited an even greater ability to evade immunity induced not only by vaccination and prior infection but also by therapeutic monoclonal antibodies.^20–25^ Omicron subvariants subsequently evolved into multiple sublineages, including early BA.5 and its sublineages,^26^ which dominated in mid-2022, followed by highly immune-evasive XBB sublineages in mid-2023,^27^ and recently, JN.1 sublineages,^28–33^ which have been driving global infection waves since mid-to-late 2024.^34^

Notably, the XBB sublineages and their descendants, including JN.1, constitute a major antigenic shift. Compared with the highly mutated Omicron variant, XBB sublineages harbor additional mutations in the spike protein and exhibit extensive resistance to neutralization by antibodies induced through vaccination and infection.^27,34^ Hence, to address waning immunity and the spread of XBB sublineages in various countries, including China, XBB variant-specific vaccines have been developed and deployed as booster doses for previously infected or vaccinated individuals. Real-world and clinical trial data have shown that these XBB-containing boosters can elicit a strong nAb response against XBB.1.5 and related lineages, as well as cross-neutralization against the latter JN.1 variant, although with slightly reduced neutralization efficacy and protection against the latter.^28,31,35–38^ However, most of the existing evidence has focused mainly on widely used mRNA-based XBB-containing vaccines,^10,34,35,39–42^ whereas data on non-mRNA vaccines, particularly protein-based platforms, which are valued for their favorable safety and immunogenicity profiles, remain limited.^36,43^ Moreover, the durability of the nAb response following XBB-containing vaccines and their correlation with subsequent infections, particularly in older adults, remains poorly characterized.^39^ Previous studies have shown that older adults exhibit a weaker humoral immune response to vaccination than do younger individuals (≤65 years)^44–46^ and a more pronounced decline in the humoral immune response over time.^47^ Nevertheless, the durability of the nAb response to XBB-containing vaccines in older adults remains unknown.

To address this gap, we recently conducted a head-to-head comparison of nAb responses induced by three protein-based XBB-containing vaccines—the trivalent XBB.1.5 (Tri‑XBB.1.5) vaccine, bivalent Omicron XBB (Bi‑Omi‑XBB) vaccine, and tetravalent XBB.1 (Tetra‑XBB.1) vaccine—in adults aged >65 years.^43^ Our initial findings, assessed three weeks post-vaccination, revealed that these three XBB-containing vaccines significantly increased both the potency and breadth of nAbs against not only XBB.1.5 but also the variants JN.1, KP.2, and KP.3.^43^ While the WHO global vaccine advisory has now recommended a shift to JN.1-lineage boosters for 2024‒2025,^48^ evaluating the durability of nAb responses induced by XBB-containing vaccines remains critically important. These data provide essential insights into the longevity of bridge neutralization against variants that circulated during the study period. In this study, we report a 6-month prospective follow-up of our previous cohort of 90 older adults who received one of the three protein-based XBB-containing booster vaccines.^43^ We longitudinally assessed the kinetics of nAb against a panel of SARS-CoV-2 variants, which included previously tested ancestral D614G and BA.5, XBB.1.5, and JN.1 variants, as well as the additional JN.1 subvariants KP.2, KP.3, KP.3.1.1, and XEC, which were identified and prevalent in China during our 6-month follow-up (April–October 2024) according to the data from the Chinese Center for Disease Control and Prevention.^49^ In addition, we compared the nAb titers elicited by the three XBB-containing vaccines and examined SARS-CoV-2 infections that occurred during the 6-month follow-up period, including both laboratory-confirmed and serologically inferred cases, to explore the relationship between antibody maintenance and the risk of infection in this high‑risk age group.

## Results

### Study participants

**Fig. 1:**
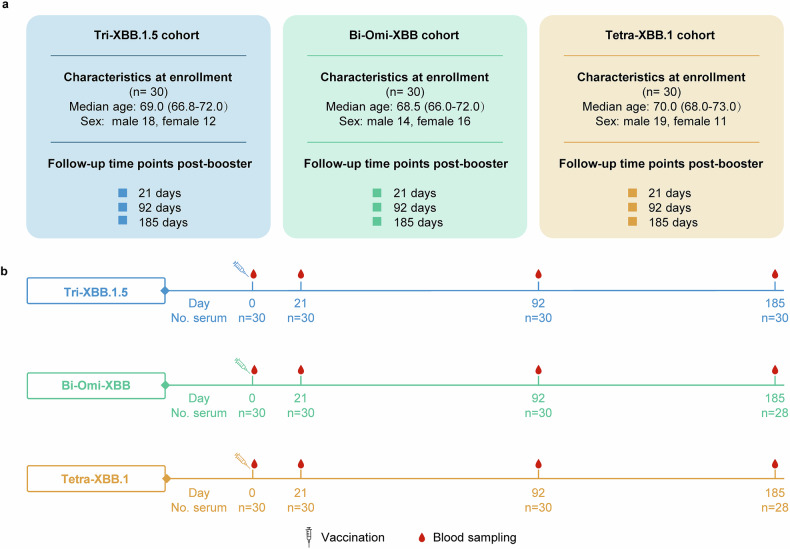
Study design and longitudinal sample collection for XBB-containing vaccine booster cohorts. **a** Enrollment and demographics of the three cohorts receiving the Tri‑XBB.1.5 vaccine, Bi‑Omi‑XBB vaccine, and Tetra‑XBB.1 vaccine. Follow-ups were conducted at 21, 92, and 185 days post-booster vaccination to evaluate the durability of neutralizing antibodies and SARS-CoV-2 infections. **b** Timeline of booster vaccination and serum sample collection for the three cohorts. Longitudinal blood sampling for each cohort was conducted at baseline (day 0) and post-booster (days 21, 92, and 185). The Tri‑XBB.1.5 cohort maintained complete sample availability (*n* = 30 per time point), and the Bi‑Omi‑XBB and Tetra‑XBB.1 cohorts maintained complete sample (*n* = 30) availability at days 0, 21, and 92 but 28 on day 185 post-booter vaccination. The syringe indicates the administration of booster vaccination; the red raindrop represents blood collection. The days associated with vaccination and blood sampling are labeled below the corresponding time points, and the serum samples are labeled below the days

In April 2024, we conducted a prospective cohort study of 90 older adults aged >65 years who received XBB-containing protein vaccines.^43^ The participants were randomly assigned (1:1:1) to receive one of three single-dose booster regimens—the Tri‑XBB.1.5 vaccine, Bi‑Omi‑XBB vaccine, or Tetra‑XBB.1 vaccine—with 30 participants allocated to each cohort. The baseline demographic characteristics, including age, sex, medical conditions, body mass index, and pre-booster antibody titers against the tested variants, were well balanced across the three cohorts (Fig. 1a) and have been described in detail in our previous publication.^43^

The serum samples were collected at four time points—baseline (day 0) and days 21, 92, and 185 post-booster vaccination (Fig. 1a). The Tri‑XBB.1.5 cohort maintained complete sample availability at all time points (*n* = 30), whereas the Bi‑Omi‑XBB cohort and the Tetra‑XBB.1 cohort retained 28 participants on day 185 (Fig. 1b). nAb titers at baseline and day 21 post-booster vaccination have been previously reported.^43^ In the present report, we provide new data from days 92 and 185 post-booster vaccination alongside the day 21 data from our previous study for longitudinal comparison and assessment of temporal trends.^43^

### Confirmed and probable SARS-CoV-2 infections during the 6-month follow-up

During the six-month follow-up, 11 participants (12.2%) experienced symptomatic SARS-CoV-2 infections confirmed by rapid antigen testing. No symptomatic infections were reported within the first 21 days post-booster vaccination. Among the 11 confirmed participants, 6 (54.5%) reported a SARS-CoV-2 infection between 43 and 91 days post-booster vaccination (median day 58), referred to as early-phase infection, and 5 (45.5%) reported an infection between 117 and 185 days post-booster vaccination (median day 165), referred to as late-phase infection. All confirmed symptomatic infections were mild to moderate, with symptom resolution occurring within 5‒7 days, and the patients did not require hospitalization. All infections were accompanied by at least one symptom (Table 1). The most common symptoms were cough (63.6%), fatigue (27.3%), sore throat (45.5%), nasal congestion (36.4%), and runny nose (18.2%). In addition, more infections (6, 54.5%) occurred in the Bi‑Omi‑XBB.1 cohort, followed by the Tetra‑XBB.1 cohort (3, 27.3%) and the Tri‑XBB.1.5 cohort (2, 18.2%) (Table 1).

**Table 1:** Demographic and clinical characteristics of confirmed infections by rapid antigen testing

Individual No.	Vaccine type for booster	Age	Sex	Date of symptom onset	Interval days between vaccination and infection^a^	Symptoms
A005	Trivalent XBB.1.5 vaccine	69	Male	10/15/2024	170	Cough
A030	Trivalent XBB.1.5 vaccine	71	Male	8/17/2024	111	Sore throat, nasal congestion, and dizziness
B031	Bivalent Omicron XBB vaccine	67	Male	7/28/2024	91	Fatigue
B036	Bivalent Omicron XBB vaccine	70	Female	6/10/2024	43	Cough
B037	Bivalent Omicron XBB vaccine	66	Female	6/19/2024	52	Sore throat and runny nose
B044	Bivalent Omicron XBB vaccine	66	Female	8/22/2024	116	Cough and sore throat
B059	Bivalent Omicron XBB vaccine	66	Female	7/22/2024	85	Cough, fatigue, and nasal congestion
B060	Bivalent Omicron XBB vaccine	68	Female	6/15/2024	48	Sore throat and nasal congestion
C061	Tetravalent XBB.1 vaccine	74	Male	7/1/2024	64	Cough, sore throat, fatigue, and nasal congestion
C064	Tetravalent XBB.1 vaccine	68	Male	10/18/2024	173	Cough, headache, and runny nose
C071	Tetravalent XBB.1 vaccine	72	Female	10/10/2024	165	Cough

^a^Interval days were calculated from XBB booster administration to the first antigen-positive test

In addition to confirmed symptomatic infections, 16 probable infections were identified through serological evidence, defined as elevated nAb titers at a later time point compared with an early time point (e.g., higher titers on day 92 versus day 21 or day 185 versus day 92). Among these probable patients, 10 experienced early-phase infections, and 6 experienced late-phase infections. Four probable infections (25.0%) occurred in the Tri‑XBB.1.5 cohort, and 6 each (37.5% per cohort) occurred in the Bi‑Omi‑XBB and Tetra‑XBB.1 cohorts (Table 2). When confirmed and probable infections were combined, a relatively low number of infections in the Tri‑XBB.1.5 cohort was observed compared with that in the other two cohorts; however, statistical analysis revealed no significant differences in infection rates among the three cohorts (*p* > 0.05).

**Table 2:** Demographic characteristics of participants with probable infections

Individual ID	Vaccination	Age	Sex	Probable infection period post-vaccination
A004	Trivalent XBB.1.5	71	Male	22‒92
A009	Trivalent XBB.1.5	74	Male	93‒185
A016	Trivalent XBB.1.5	69	Female	22‒92
A026	Trivalent XBB.1.5	72	Female	22‒92
B032	Bivalent Omicron XBB	67	Male	22‒92
B040	Bivalent Omicron XBB	72	Female	22‒92
B042	Bivalent Omicron XBB	71	Male	22‒92
B043	Bivalent Omicron XBB	68	Female	93‒185
B048	Bivalent Omicron XBB	71	Female	22‒92
B051	Bivalent Omicron XBB	75	Male	22‒92
C066	Tetravalent XBB.1	66	Female	93‒185
C069	Tetravalent XBB.1	77	Male	93‒185
C073	Tetravalent XBB.1	72	Male	93‒185
C078	Tetravalent XBB.1	68	Male	93‒185
C086	Tetravalent XBB.1	67	Female	22‒92
C088	Tetravalent XBB.1	67	Male	22‒92

### Dynamics of neutralizing antibodies in uninfected older adults six months after booster

**Fig. 2:**
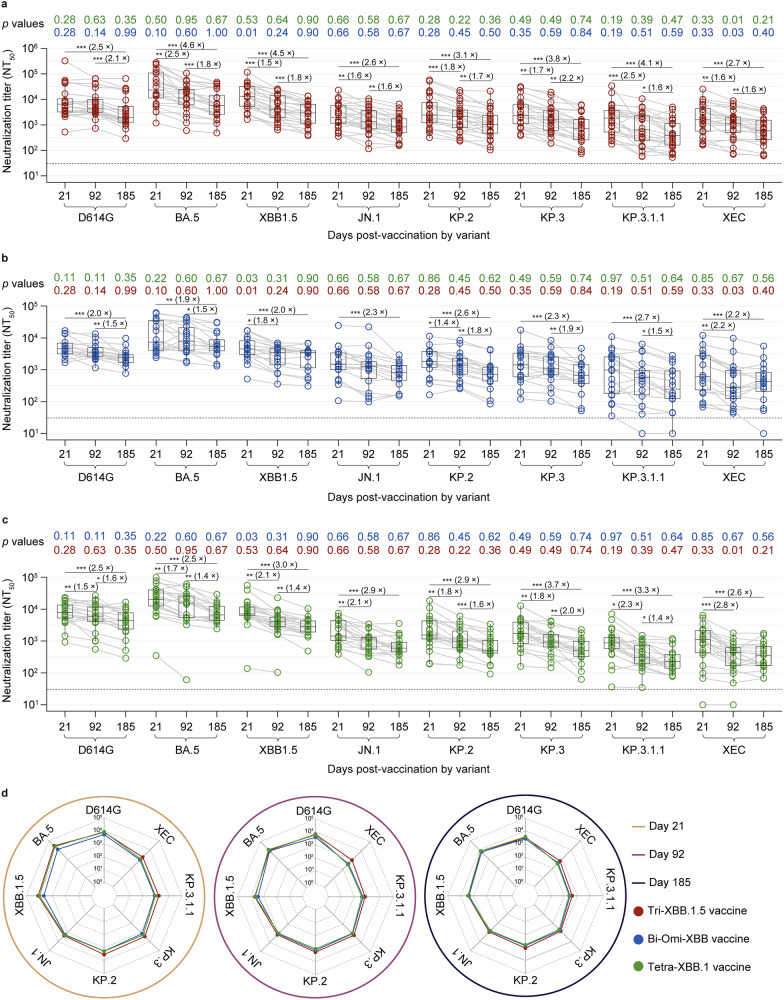
Dynamics of pseudovirus neutralization profiles in uninfected older adults six months after booster vaccination. **a**–**c** The longitudinal dynamics of 50% pseudovirus neutralization titers (NT_50_) against SARS-CoV-2 variants—D614G, BA.5, XBB.1.5, JN.1, KP.2, KP.3, KP.3.1.1, and XEC—were assessed in uninfected older adults during a six-month follow-up period after receiving a booster dose of either the Tri‑XBB.1.5 vaccine (**a**), Bi‑Omi‑XBB vaccine (**b**), or Tetra‑XBB.1 vaccine (**c**). **d** A radar plot was generated to illustrate the tropism and breadth of neutralizing antibody responses induced by the Tri‑XBB.1.5 vaccine, Bi‑Omi‑XBB vaccine, and Tetra‑XBB.1 vaccine across the tested variants during the six-month follow-up. In the boxplots (**a**–**c**), horizontal bars and boxes represent the median and interquartile range (IQR) NT_50_, respectively; whiskers reference 95% confidence intervals (CIs). The gray circles represent individual data points, with connecting lines representing longitudinal samples from the same participants. The dotted line represents the assay detection threshold (NT_50_ = 30). The fold change in geometric mean titers (GMTs) between time points is indicated in brackets. In the radar plots, the vertical line represents each variant or spoke, and the spokes are evenly distributed around the circle. Each horizontal line along a vertical spoke represents the GMT at a tenfold dilution, with the value closest to the center being 1 (10^0^) and farthest from the center being 100,000 (10^5^). A two-sided Friedman test with false discovery rate (FDR) correction was employed for within-vaccine longitudinal comparisons (three time points per vaccine). A two-tailed Kruskal‒Wallis test with the FDR was used to compare differences in neutralizing antibody titers among vaccines at different time points. A *p* value less than 0.05 was considered statistically significant. *P* values for the differences between neutralization titers across different time points for each vaccine are denoted by asterisks (*p < 0.05, **p < 0.01, ***p < 0.001) in (**a**), (**b**), and (**c**), and only significant differences are displayed in the figure. *P* values for comparisons between vaccines at each time point are color-coded and shown at the top in (**a**), (**b**), and (**c**). NT_50_ values are derived from a single experiment with two technical replicates

We next longitudinally assessed nAb titers against ancestral D614G and BA.5, XBB.1.5, JN.1, KP.2, KP.3, KP.3.1.1, and XEC variants in uninfected older adults via a pseudovirus neutralization assay. In all three cohorts, nAb titers against all tested variants peaked by day 21, followed by similar antibody decline kinetics over 185 days (Fig. 2). By day 92, a significant decrease in nAb titers was observed. In the Tri‑XBB.1.5 cohort, geometric mean titers (GMTs) decreased 1.5‒2.5-fold for BA.5, XBB.1.5, JN.1, KP.2, KP.3, KP.3.1.1, and XEC variants but remained largely unchanged for D614G. In the Bi‑Omi‑XBB cohort, the GMTs decreased 1.4‒2.2-fold for the XBB.1.5, KP.2, and XEC variants, whereas the titers against D614G, BA.5, JN.1, KP.3, and KP.3.1.1 showed no significant reduction. In the Tetra‑XBB.1 cohort, the GMTs decreased 1.5‒2.8-fold against all the tested variants compared with those on day 21.

By day 185, the GMTs against all tested variants further declined, with cumulative reductions from day 21 reaching 2.5–4.6-fold for the Tri‑XBB.1.5 cohort, 1.9–2.7-fold for the Bi‑Omi‑XBB cohort, and 2.5–3.7-fold for the Tetra‑XBB.1 cohort (Fig. 2a–c**)**. Compared with GMTs on day 92, there were significant reductions of 1.6–2.2-fold for all the tested variants in the Tri‑XBB.1.5 cohort; 1.5–1.9-fold for D614G, BA.5, KP.2, KP.3, and KP.3.1.1 in the Bi‑Omi‑XBB cohort; and 1.4–2.0-fold for D614G, BA.5, XBB.1.5, KP.2, KP.3, and KP.3.1.1 in the Tetra‑XBB.1 cohort (Fig. 2a‒c). Notably, neutralization of KP.3.1.1 and XEC declined markedly in all three cohorts by day 185. The GMTs against KP.3.1.1 decreased to 409 (95% confidence interval [CI]: 238–704), 277 (95% CI: 120–637), and 238 (95% CI: 160–353) for the Tri‑XBB.1.5, Bi‑Omi‑XBB, and Tetra‑XBB.1 cohorts, respectively. Similarly, GMTs against XEC decreased to 599 (95% CI: 352–1022), 364.6 (95% CI: 159–837), and 319 (95% CI: 203–502), highlighting more significant immune evasion by these two variants (Fig. 2a–c).

Further comparison of nAb titers at days 21, 92, and 185 post-booster vaccination revealed that nAb titers were comparable across the three cohorts (Fig. 2a–c). However, the Tri‑XBB.1.5 cohort exhibited relatively high and broad neutralization against all the tested variants over the 6-month follow-up compared with the Bi‑Omi‑XBB and tetravalent XBB cohorts (Fig. 2a–c). The radar plot also revealed a decline in nAb levels over time (Fig. 2d). Despite this overall decline in nAb levels over time, the Tri‑XBB.1.5 cohort maintained superior titers and neutralizing activity against the tested variants compared with the other two cohorts (Fig. 2d). Overall, nAb waning patterns were similar across XBB-containing boosters; however, KP.3.1.1 and XEC exhibited the most substantial reductions in cross-neutralization over time, highlighting the need for regular antigenic updates in vaccine formulations to address antibody waning and improve protection against evolving and highly immune-evasive SARS-CoV-2 lineages.

### Neutralizing antibodies in infected older adults six months after booster

**Fig. 3:**
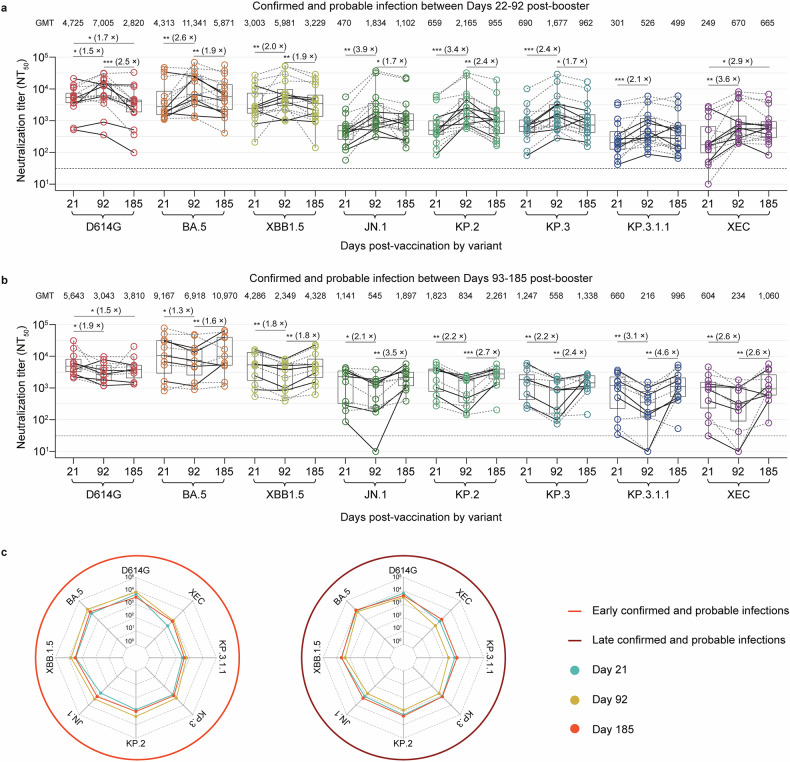
Tempro-neutralizing antibody profiles in early- and late-phase infected older adults during the six-month follow-up after booster vaccination. **a**, **b** The dynamics of 50% pseudovirus neutralization titers (NT_50_) against D614G, BA.5, XBB.1.5, JN.1, KP.2, KP.3, KP.3.1.1, and XEC were evaluated in older adults with SARS-CoV-2 infection during the early phase (days 21‒92, **a**) and late phase (days 93‒185, **b**) after receiving one of three booster vaccines, the Tri‑XBB.1.5 vaccine, the Bi‑Omi‑XBB vaccine, or the Tetra‑XBB.1 vaccine. **c** A radar chart was generated from the log_10_ GMT data to reveal the tropism of the neutralizing antibody response in participants with early and late SARS-CoV-2 infections after receiving booster vaccinations. Boxplots in **a** and **b** display the median (center line), interquartile range (box), and 95% confidence intervals (whiskers). The gray circles represent individual data points, with connecting lines representing longitudinal samples from the same participants. The neutralizing geometric mean titers (GMTs) are shown at the top in (**a**) and (**b**). The dotted line represents the assay detection threshold (NT_50_ = 30). The fold change in the GMTs between time points is indicated in brackets. In the radar plots, the vertical line represents each variant or spoke, and the spokes are evenly distributed around the circle. Each horizontal line along a vertical spoke represents the GMT at a tenfold dilution, with the value closest to the center being 1 (10^0^) and farthest from the center being 100,000 (10^5^). Lines connecting the values reflect the neutralization landscape induced by each vaccine across the tested variants. A two-sided Friedman test with the false discovery rate method for multiple comparisons was applied to evaluate differences in NT_50_ values among viral variants at different time points within each vaccine group. A *p* value less than 0.05 was considered statistically significant, and only significant differences are displayed in the figure. *P* values are denoted as *p < 0.05, **p < 0.01, and ***p < 0.001. The neutralization assay was run in a single experiment with two technical replicates

We next analyzed the nAb responses of participants with confirmed (*n* = 11) or probable (*n* = 16) SARS-CoV-2 infections. Owing to the limited number of participants with SARS-CoV-2 infections per vaccine cohort, all infections (confirmed and probable) were pooled and grouped according to the early phase (days 22‒92) or late phase (days 93‒185). In the early-phase-infected participants (confirmed: *n* = 6, probable: *n* = 10), the GMTs on day 92 exhibited a 1.5‒3.9-fold increase compared with the GMTs on day 21 (Fig. 3a), reflecting post-infection immune boosting. By day 185, the GMTs significantly declined 1.2‒2.5-fold relative to those on day 92 post-booster vaccination, returning to levels comparable to those on day 21. Notably, the GMTs against the KP.3.1.1 and XEC variants were comparable to the GMTs on day 92. In late-phase-infected participants (confirmed: *n* = 5, probable: *n* = 6), GMTs on day 92 showed a 1.3‒3.1-fold reduction from day 21 (Fig. 3b) but subsequently increased 1.3‒4.6-fold by day 185 compared with day 92, stabilizing at levels similar to those on day 21. More intuitively, the radar plot revealed that both early-phase and late-phase infections presented broader and more balanced neutralizing activity against the tested variants than did GMTs on day 21 post-booster vaccination, and both achieved sustained nAb elevation comparable to pre-infection baselines (Fig. 3c). However, early-phase infections result in transient immune boosting followed by waning, whereas late-phase infections result in decreased but durable antibody recovery. These findings suggest that SARS-CoV-2 infection enhances the breadth of cross-variant neutralization.

### Neutralizing antibody titers and SARS-CoV-2 infection

**Fig. 4:**
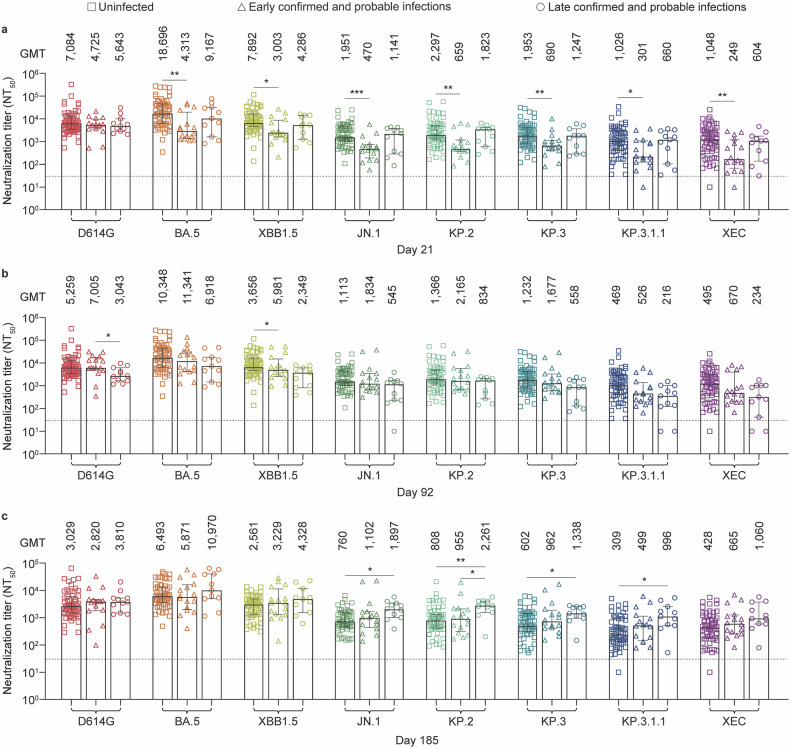
Comparative analysis of neutralizing antibody responses in uninfected and infected older adults after booster vaccination. **a**‒**c** Neutralizing titers against the indicated pseudoviruses in sera collected from uninfected (squares), early infected (infected between days 22‒92, triangles), and later infected (infected between days 93‒185, circles) participants at days 21 (**a**), 92 (**b**), and 185 (**c**) after one-dose booster of the Tri‑XBB.1.5, Bi‑Omi‑XBB, or Tetra‑XBB.1 vaccine. Each square, triangle, or circle represents the individual the 50% neutralization titer (NT_50_). The vertical bars represent the median and 95% confidence intervals. The geometric mean titers (GMTs) are shown above each group. The dotted line represents the assay detection threshold (NT_50_ = 30). Statistical comparisons among the three groups were conducted via the Kruskal‒Wallis test with the false discovery rate. A *p*-value less than 0.05 was considered statistically significant, and only significant differences are displayed in the figure. *P*-values are denoted as *p < 0.05 and **p < 0.01

To explore the relationship between nAb titers post-booster vaccination and subsequent SARS-CoV-2 infection, we first compared GMTs against D614G and BA.5, XBB.1.5, JN.1, KP.2, KP.3, KP.3.1.1, and XEC variants on day 21 post-booster vaccination among uninfected participants, early-phase-infected participants (days 22‒92), and late-phase-infected participants (days 93‒185). On day 21, compared with uninfected participants, early-phase-infected participants had significantly lower GMTs against the tested variants (*p* < 0.05), except for D614G (Fig. 4a). In contrast, the GMTs of late-phase-infected participants were comparable to those of uninfected participants. When the GMTs on day 92 post-booster vaccination were compared, no significant differences were observed between late-phase-infected and uninfected participants (Fig. 4b). By day 185, late-phase-infected participants presented greater GMTs against JN.1 (*p* = 0.01), KP.2 (*p* = 0.008), KP.3 (*p* = 0.046), and KP.3.1.1 (*p* = 0.02) than uninfected participants did, whereas GMTs against the other tested variants were comparable across the three cohorts (Fig. 4c). Notably, the GMTs on day 185 did not differ significantly between the early- and late-phase-infected participants. These findings suggest that individuals with lower initial nAb titers post-booster vaccination correlate with early-phase infections, whereas late-phase infections are likely due to waning nAb, underscoring the importance of updated booster strategies to compensate for waning immunity against newly emerging and highly immune-evasive variants.

### Antigenic cartography

**Fig. 5:**
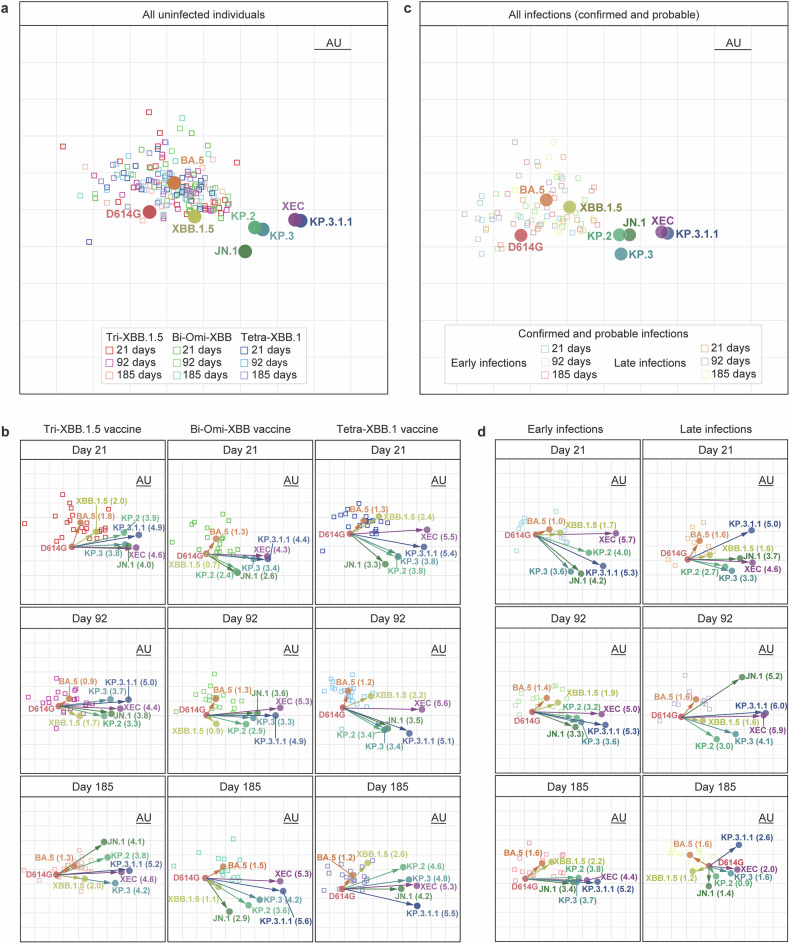
Antigenic map of serum virus neutralization data. Antigenic maps were generated via the Racmacs program (1.1.4) on the basis of neutralization titers against the indicated pseudoviruses. **a**, **c** Maps derived from all vaccine cohorts after vaccination. **b** Maps of SARS-CoV-2-uninfected older adults at days 21, 92, and 185 after receiving the Tri‑XBB.1.5 vaccine, Bi‑Omi‑XBB vaccine, or Tetra‑XBB.1 vaccine, during six months of follow-up. **d** Maps of SARS-CoV-2-infected older adults 92 or 185 days after receiving booster vaccination during six months of follow-up. In each map, circles represent the indicated variants, and squares denote individual serum samples. Both the x- and y-axes are in antigenic units (AUs), where one grid corresponds to a 2-fold serum dilution of the neutralization titer. One square corresponds to one AU squared. Arrows between D614G and selected variants indicate the antigenic distance in AUs

We next generated two-dimensional antigenic maps via neutralization data from uninfected, early-phase-infected (days 22‒92), and late-phase-infected (days 93‒185) participants to visually summarize our findings of nAb titers and explore the antigenic relationships between variants, where distances represent neutralization differences between variants. In uninfected participants, the antigenic map revealed that the BA.5, XBB.1.5, and JN.1 variants are antigenically distant, the variants KP.2 and KP.3 are grouped closely but antigenically distant from the JN.1 variant, and the KP.3.1.1 and XEC variants are grouped closely but are positioned farthest from D614G and distant from KP.2 and KP.3 (Fig. 5a). The serum samples from all three time points (21, 92, and 185 days) after booster vaccination largely overlapped and were positioned around BA.5 and XBB.1.5 (Fig. 5a). However, serum samples from early time points (22‒92 days) shifted antigenically toward JN.1, KP.2, and KP.3, whereas serum samples from later time points (93‒185 days) drifted back toward BA.5 and XBB.1.5. When analyzed by vaccine type, the antigenic distances between the ancestral D614G and Omicron subvariants were similar across all time points, but Tri‑XBB.1.5 vaccination relatively reduced the antigenic distance compared with Bi‑Omi‑XBB or Tetra‑XBB.1 vaccination, suggesting broader antibody potency and cross-variant coverage in the Tri‑XBB.1.5 cohort (Fig. 5b).

In participants with confirmed or probable SARS-CoV-2 infections, the overall antigenic patterns were similar to those in uninfected participants (Fig. 5c). For early-phase-infected participants, antigenic distances on day 92 were marginally reduced compared with those in the uninfected individuals but converged by day 185 (Fig. 5d, left panel). In contrast, later-phase infected participants presented a significant reduction in the distance between D614G and the tested variants of approximately 2 antigenic units (AUs) by day 185 (Fig. 5d, right panel), which highlights the influence of the timing of infection or infection by a distinct viral strain on immune responses to emerging variants. Taken together, these results suggest that both the timing of infection and the infecting viral strain, as well as the vaccine type, likely play pivotal roles in determining the breadth and potency of nAb responses.

## Discussion

Since older adults are particularly susceptible to severe COVID-19, understanding the immune response in this population is critical for optimizing vaccination strategies. In this study, we systematically evaluated the dynamics of nAb responses and SARS-CoV-2 infections over six months in older adults aged >65 years following booster vaccination with Tri‑XBB.1.5, Bi‑Omi‑XBB, or Tetra‑XBB.1. By assessing the nAb response over a six-month follow-up, the results provide valuable insights into the durability of XBB-containing vaccine-induced nAb response, SARS-CoV-2 infections, and the performance of these vaccines against SARS-CoV-2 variants, including the immune-evasive variants KP.3.1.1 and XEC.

All three XBB-containing vaccines in this study elicited strong nAb responses against D614G and all the tested variants, including the recently emerged KP.3.1.1 and XEC variants, at 21 days post-booster vaccination. This finding aligns with previous studies showing that updated XBB-containing vaccines retain substantial immunogenicity against antigenically divergent variants, although with reduced potency compared with ancestral strains.^31,35,50^ We observed a gradual decline in nAb responses over time, yet responses remained detectable and relatively durable for up to six months. Similar durability has been reported for trivalent XBB.1.5 protein-based vaccines in mice, which provide broad protection and maintain humoral and cellular immunity for approximately 210 days.^50^

In contrast, recipients of an XBB.1.5 monovalent mRNA booster exhibited minimal or only slight waning of nAb titers against XBB.1.5, D614G, BA.5, and JN.1 variants over six months.^39^ Moreover, GMTs against D614G (12,762 vs.3,029), BA.5 (15,338 vs.6,493), XBB.1.5 (3,224 vs.2,561), and JN.1 (1,329 vs.760) were substantially greater six months after mRNA boosting than those observed in our cohort following XBB-specific protein-based vaccination. While these differences may suggest that protein-based vaccines generate less durable neutralizing responses than do mRNA platforms, such cross-study comparisons should be interpreted with caution, given differences in participant demographics, prior immunity, and assay methodologies.

Notably, we observed a 2.5‒4.6-fold reduction in nAb against the newer KP.3.1.1 and XEC variants by day 185. The substantial decline in neutralizing activity against the newer KP.3.1.1 and XEC variants indicated an arms race between the ongoing evolution of SARS-CoV-2 and vaccine-induced immunity, which supports the WHO’s updated recommendations for 2024‒2025 boosters and highlights the short-term durability of neutralizing immunity induced by the nonvariant-specific vaccine against the newly emerged variants.

The significant decline in nAb titers against newer variants also suggests that the antigenic distance from the vaccine strain plays an important role in determining durability, with more distinct variants evading cross-reactive nAbs more rapidly. This situation demands agile vaccine policies, updated variant-specific vaccines, or shorter booster intervals (e.g., 4‒6 months) to mitigate the impact of antibody waning, especially against the emergence of highly immune-evasive variants that harbor additional spike protein mutations, as recommended by global surveillance initiatives.^48^ On the basis of our findings, administering a second booster approximately six months after the prior dose is recommended to maintain adequate protection in older adults, with priority given to updated, variant-specific vaccines where available. While mRNA vaccines may provide longer-lasting immunity,^39^ protein-based vaccines remain an important alternative in settings where mRNA platforms are inaccessible. In summary, a six-month booster—preferably with a variant-adapted vaccine—can help sustain protection in vulnerable populations, especially older adults, in the face of ongoing SARS-CoV-2 evolution.

For SARS-CoV-2 infection during the six-month follow-up, symptomatic SARS-CoV-2 infections were recorded in 12.2% of the participants, and probable infections were recorded in an additional 17.8%, which is consistent with global observations of reduced vaccine effectiveness against Omicron sublineages, particularly the XBB linages and JN.1.^10,40–42^ Notably, all symptomatic infections experienced mild to moderate illness and did not require hospitalization, suggesting the effectiveness of XBB-containing vaccines in mitigating severe disease despite being unable to prevent infection due to waning nAb levels. Although not statistically significant, the Tri‑XBB.1.5 cohort presented a relatively low SARS-CoV-2 infection rate compared with the other two cohorts. Several factors may contribute to the increased nAb responses and relatively low infection rates observed with the trivalent vaccine. Compared with the Bi‑Omi‑XBB and Tetra‑XBB.1 vaccines, the Tri‑XBB.1.5 vaccine contained a 33% greater antigen dose for the XBB.1.5 variant, which more closely matched the antigens of the circulating strains and may reduce antigenic competition. However, the potential advantages of the trivalent vaccine require confirmation in larger cohorts to determine whether they translate into clinical protection. On the other hand, we also observed that early-phase infections were associated with a lower peak nAb titer (day 21 post-booster vaccination), highlighting the role of higher peak nAb levels in preventing early infections. In contrast, late-phase infections likely reflect a combination of a decline in nAb over time and the emergence of variants that are more resistant to neutralization, such as KP sublineages, that circulated during the study period. Together, these findings suggest that the development of vaccines targeting current circulating variants or shortened booster intervals would be valuable for maintaining high nAb levels and reducing infection risk.

Our study also provides an understanding of the dynamic interaction between immunity elicited by infections and that induced through vaccination. We found that early-phase infections triggered transient antibody increases (1.5‒3.9-fold increases by day 92), followed by rapid decreases to near-baseline levels by day 185. This short-lived boosting effect is consistent with observations in mRNA vaccine studies, where post-infection antibody surges are often short-lived,^51^ likely due to limited germinal center engagement in older adults.^44,52^ Conversely, late-phase infections (days 93‒185) elicited stronger and more sustained immune stimulation, with 1.3‒4.6-fold increases in nAb titers by day 185 compared with day 92, and enhanced the neutralization activity to specific variants, including the JN.1 and KP sublineages. Antigenic cartography further revealed that late-phase infections were associated with a significant reduction in antigenic distances—approximately 3 antigenic units—between D614G and the tested variants. Such immunological benefits during the waning phase may be attributed to exposure to distinct or newer viral strains, as XDV and its subvariants were the dominant variants in China since July 2024; these variants may induce strong germinal center B-cell responses and can generate de novo B-cell responses targeting variant-specific epitopes, as previously observed in Omicron boosting vaccination in humans.^53^

We also observed that by day 185, participants infected late had higher nAb titers than those who remained uninfected, largely due to the recent post-infection boost. In contrast, those infected early initially exhibited increased antibody levels after infection, but their titers decreased to levels similar to those in the late-infected group by day 185, which is consistent with typical post-infection antibody kinetics. This pattern suggests that early infections likely occurred in individuals with suboptimal peak vaccine responses, whereas late infections were more often driven by antibody waning and the circulation of more immune-evasive variants. Overall, regardless of early or late infection status, nAb levels 185 days post-booster vaccination were comparable to pre-infection levels, reaffirming that infection alone cannot replace vaccine boosting.

This study has several limitations. First, the modest sample size limits the statistical power to detect significant differences between vaccine groups, particularly for infection incidence. The small number of infection events also precluded robust statistical comparisons, multivariate adjustment of infection risk, or definitive conclusions regarding clinical protection. Although serostatus and prior infection were comparable at baseline, durability analyses were not stratified by these variables due to reduced power in subgroup analyses. Second, the current analysis was restricted to nAb responses, as the primary aim was to evaluate nAb durability against emerging variants. T-cell responses, which may be critical in preventing severe disease, have not yet been presented in this report; however, our one-year longitudinal follow-up will address this gap, including the use of scRNA-seq to characterize cellular immune dynamics. In addition, although investigations are underway, kinetic analyses of the memory B-cell response are not available to further differentiate the durability of the nAb response elicited by the three XBB-containing vaccines. Third, there are several established methods for confirming SARS-CoV-2 infection, including molecular diagnostic methods such as RT‒PCR, antigen detection, virus isolation and culture, and serological assays. In this study, we used symptom-based surveillance complemented by serological analysis, which may have underestimated asymptomatic or mild infections lacking influenza-like illness symptoms and may have missed infection in participants with attenuated or rapidly waning antibody responses. Additionally, the lack of systematic virological screening (e.g., routine PCR testing) and viral sequencing precluded the precise identification of causative variants or the confirmation of infection timing. Future studies should benefit from scheduled PCR testing irrespective of symptoms, when feasible, to improve infection confirmation. Finally, these results may not apply to other vaccine platforms that are widely deployed in other countries.

In conclusion, this longitudinal study revealed that three protein-based XBB-containing vaccines elicit improved and broadly cross-protective nAb responses against diverse SARS-CoV-2 variants in older adults. Nonetheless, the nAb titers wane considerably over time, particularly against newly emerged KP.3.1.1 and XEC variants. These findings underscore the need to update booster strategies and formulations to match circulating or future strains, optimizing booster intervals. However, XBB-containing protein-based vaccines remain a valuable component of the COVID-19 vaccine portfolio, particularly in countries where mRNA vaccines are less accessible, and their strategic deployment could help sustain protection in vulnerable populations.

## Materials and methods

### Study population and follow-up

In April 2024, we enrolled a cohort of 90 adults older than 65 years to receive a booster dose of one of the Tri-XBB.1.5, Bi-Omi-XBB, or Tetra-XBB.1 vaccines to evaluate nAb responses against ancestral D614G and variants BA.5, XBB.1.5, JN.1, KP.2, and KP.3.^43^ The detailed eligibility criteria, consent processes, randomization, characteristics of the cohort, and nAb response before and three weeks post-booster were also described in our previous publication.^43^ This study aims to describe the kinetics of nAbs after vaccination. The cohort participants were further followed up 3 and 6 months after vaccination. The sample size was not determined by statistical methods but was based on the availability of vaccine doses of three types of vaccines at the study site. Vaccine assignment was randomized but not blinded to the investigators.

The study was conducted in accordance with the Declaration of Helsinki and was approved by the ethical committee of the Academy of Military Medical Sciences (AF/SC-08/02.197). Written informed consent and completion of the demographic questionnaire were obtained from each participant at enrollment.

### Vaccines for booster vaccination

As described in our previous study,^43^ the Tri‑XBB.1.5 30 μg vaccine contains three proteins incorporating the receptor binding domain and heptad repeat motifs from the Delta, BA.5, and XBB.1.5 spike glycoproteins. The Bi‑Omi‑XBB 20 μg vaccine includes two recombinant proteins derived from the ancestral SARS-CoV-2 (Wuhan-Hu-1) strain and the Omicron XBB variant. The Tetra‑XBB.1 30 μg vaccine contains four spike extracellular domain trimers from the Beta, Delta, BA.1, and XBB.1 variants. The Tri‑XBB.1.5 vaccine was administered intramuscularly at 30 μg in a 0.25 mL volume, whereas the Bi‑Omi‑XBB and Tetra‑XBB.1 vaccines were administered intramuscularly at 20 and 30 μg in a 0.5 mL volume, respectively.

### Cell lines

HEK-293T cells (ATCC, CRL-3216) were authenticated by the supplier (Certificate of Analysis) and confirmed to be free of mycoplasma contamination. HEK-293T cells were maintained at 37 °C with 5% CO_2_ in Dulbecco’s modified Eagle’s medium (DMEM, Gibco), supplemented with 10% (v/v) heat-inactivated fetal bovine serum (FBS, Gibco) and 1% penicillin-streptomycin (Gibco). The cells were passaged every 48‒72 h upon reaching confluence via 0.25% trypsin with 1 mM EDTA (Solarbio). HEK-293T cells stably expressing human ACE2 (HEK-293T-hACE2) were grown under the same culture conditions.

### Spike plasmid pseudovirus production

Pseudoviruses were generated by cotransfecting HEK-293T cells with a human immunodeficiency virus backbone plasmid encoding firefly luciferase (pNL4-3-R-E-luciferase) and a pcDNA3.1 vector encoding the spike proteins of D614G, XBB.1.5, JN.1, KP.2, KP.3, KP.3.1.1, or XEC. These codon-optimized, full-length open reading frames were synthesized by GenScript (Nanjing, China), and all the sequences were verified via Sanger sequencing. Mutations in the spike proteins of D614G, BA.5, XBB.1.5, JN.1, KP.2, and KP.3 were described in our previous studies,^27,43,54^ and the sequence accession numbers and spike mutations for KP.3.1.1 and XEC relative to D614G are provided in Supplementary Table 1. Following transfection, the cell culture medium was replaced with fresh medium at 24 h, and the supernatants were harvested at 48 h post-transfection and clarified by centrifugation at 300 × *g* for 10 min before being aliquoted and stored at −80 °C until use. Pseudoviruses were normalized by determining infectious titers via the 50% tissue culture infectious dose (TCID_50_) before the pseudovirus neutralization assay, with titers calculated according to the Reed‒Muench method as previously described.^55^ All viral stocks were diluted to achieve equivalent TCID_50_ values across experimental conditions.

### Pseudovirus neutralization assay

A pseudovirus neutralization assay was conducted as reported previously.^27,43,54^ Briefly, heat-inactivated sera were diluted in eight 3-fold dilutions starting at 1:30 in duplicate and incubated with 500‒1000 TCID_50_ of the pseudotyped viruses for one hour at 37 °C and 5% CO_2_, followed by the addition of 1 × 10^4^ 293T-ACE2 cells per well. After 48 h at 37 °C and 5% CO_2_, the cells were lysed with passive lysis buffer (Vazyme) and luminescence was measured via luciferase assay buffer (Vazyme, China) on a GloMax 96 Microplate Luminometer (Promega). The 50% neutralization titer (NT_50_) was derived via a four-parameter nonlinear regression inhibitor curve in GraphPad Prism 9.0.0 (GraphPad Software). NT_50_ was defined as the maximum reciprocal serum dilution at which a 50% reduction in relative light units relative to the average of the virus control wells occurred. Samples with an NT_50_ < 30 (limit of detection) were considered negative and assigned a value of 10 for geometric mean titer calculation. For each assay plate, a pseudovirus-only positive control and a cell-only background control were included to ensure assay consistency and data quality.

### Antigenic cartography

An antigenic map was constructed via the antigenic cartography approach.^56^ The antigenic distances between D614G and the tested SARS-CoV-2 variants were calculated by integrating the neutralization potency of each serum sample, with these distances inversely proportional to the log2 titer of the antigens and antisera. The Racmacs package (https://acorg.github.io/Racmacs/, v.1.1.4) within R generated the map, employing 2000 optimization iterations and setting the minimum column basis parameter to “none”. The “map distances function” of the Racmacs package was used to determine antigenic distances, and the average distances for all sera to variants were utilized to represent the final distances. D614G was used as the center of the serum sample for each cohort, and the seeds for each antigenic map were manually adjusted to ensure that XEC was displayed horizontally relative to the serum.

### Surveillance of SARS-CoV-2 infection

The exploratory objective was to explore SARS-CoV-2 infection among participants during six-month follow-ups. At the 3- and 6-month follow-up visits, SARS-CoV-2 infection data, including symptoms, test results, and dates of positive tests, were collected via a questionnaire. We defined confirmed SARS-CoV-2 infection as the presence of influenza-like illness (ILI) symptoms—including fever (≥38 °C), cough, or fatigue—and yielded a positive result from a self-administered rapid antigen test (Hotgen™ COVID-19 Antigen Home Test Kit, Colloidal Gold; Beijing Hotgen Biotech Co. Ltd., China). Notably, no systematic screening for asymptomatic infections was conducted during the study period. Probable SARS-CoV-2 infection was identified in participants who did not report ILI symptoms but presented elevated nAb titers at later follow-up time points. Specifically, probable infection was defined as a ≥ 1.5-fold increase in nAb titers against at least five of the tested pseudoviruses (D614G, BA.5, XBB.1.5, JN.1, KP.2, KP.3, KP.3.1.1, or XEC) on day 92 compared with day 21 or on day 185 compared with day 92 and the absence of a positive rapid antigen test result during the follow-up period. This serological criterion aims to capture subclinical or unreported infections that may have occurred between visits. nAbs were measured via a pseudovirus neutralization assay as described above. Because previous studies have shown that anti-N antibodies persist >12 months post-infection/vaccination,^57,58^ a serological assay of anti-nucleocapsid antibodies was precluded because of prior inactivated vaccines and BA.5/BF.7 breakthrough infection and, later, additional infection exposure.

### Statistical analysis

We summarized participant demographics via a descriptive approach. For categorical variables, data are presented as counts and frequencies, and continuous variables with nonnormal distributions are expressed as medians and interquartile ranges. Differences in categorical variables among the three study groups were analyzed via the chi-square test or Fisher’s exact test. Multiple comparisons of nAb titers against the tested pseudoviruses were performed via the Friedman test for paired samples, with the false discovery rate (FDR) method. For comparisons of unpaired nAb titers between groups, the Kruskal‒Wallis test with the FDR method was employed. All the statistical analyses were performed with GraphPad Prism software (version 9.0.0; La Jolla, California, USA). All the statistical tests were 2-sided with a significance level of 0.05.

## Supplementary information

Supplementary information

## Data Availability

All data that support the results of this study are included in the main text and supplementary information. Raw data and further information are available from the corresponding author upon reasonable request.
