# Supplementary material for: Neutralizing antibody durability and SARS-CoV-2 infection in older adults six months after XBB-containing vaccine booster
Source: Signal Transduct Target Ther. 2025 Oct 9;10:336. doi: 10.1038/s41392-025-02437-y (PMC12508101; doi:10.1038/s41392-025-02437-y)
Supplement: Supplementary file 1 — Supplementary information [file 41392_2025_2437_MOESM1_ESM.docx]

Supplementary Materials for

Neutralizing antibody durability and SARS-CoV-2 infection in older adults six months after XBB-containing vaccine booster

Rui-Rui Chen, Guo-Ping Cao, Xue-Dong Song, Mei Lu, Ming-Ming Wang, Xue-Jun Wang, Meng-Fei Wang, Shuang-Qing Wang, Sheng Wan, Guo-Jian Yang, Lei Lv, Yi-Ming Ma, Yi-Man Cheng, Meng Kong, Xue-Juan He, Hai-Yan Yang, Bing-Dong Zhan, Mai-Juan Ma

Correspondence to: yhy@zzu.edu.cn; bd_zhan@126.com; mjma@163.com

**This PDF file includes:**

Table S1

Table S1. Sequence accession numbers and mutations in the spike protein of variants used in the study.

| **Variants** | **Sequence accession numbers** | **Mutations** |
| --- | --- | --- |
| **KP.3.1.1** | XOB88638 | ins16_ MPLF, T19I, R21T, L24-, P25-, P26-, A27S, S31-, S50L, H69-, V70-, V127F, G142D, Y144-, F157S, R158G, N211-, L212I, V213G, L216F, H245N, A246D, I332V, G339H, K356T, S371F, S373P, S375F, T376A, R403K, D405N, R408S, K417N, N440K, V445H, G446S, N450D, L452W, L455S, F456L, N460K, S477N, T478K, N481K, V483-, E484K, F486P, Q493E, Q498R, N501Y, Y505H, E554K, A570V, D614G, P621S, H655Y, N679K, P681R, N679K, N764K, D796Y, S939F, Q954H, N969K, V1104L, P1143L |
| **XEC** | XEQ78421 | ins16_ MPLF, T19I, R21T, T22N, L24-, P25-, P26-, A27S, S50L, F59S, H69-, V70-, V127F, G142D, Y144-, F157S, R158G, N211-, L212I, V213G, L216F, H245N, A246D, I332V, G339H, K356T, S371F, S373P, S375F, T376A, R403K, D405N, R408S, K417N, N440K, V445H, G446S, N450D, L452W, L455S, F456L, N460K, S477N, T478K, N481K, V483-, E484K, F486P, Q493E, Q498R, N501Y, Y505H, E554K, A570V, D614G, P621S, H655Y, N679K, P681R, N679K, N764K, D796Y, S939F, Q954H, N969K, V1104L, P1143L |
